# Highlights of clinical and laboratory parameters among severe COVID-19 patients treated with tocilizumab: a retrospective observational study

**DOI:** 10.1590/1516-3180.2021.0604.R1.23112021

**Published:** 2022-07-15

**Authors:** Melahat Uzel Şener, Tuğba Çiçek, Ayperi Öztürk

**Affiliations:** I MD. Physician, Pulmonary Medicine Department, Health Sciences University Faculty of Medicine, Atatürk Chest Diseases and Thoracic Surgery Training and Research Hospital, Ankara, Turkey.; II MD. Physician, Pulmonary Medicine Department, Konya Numune Hospital, Konya, Turkey.; III MD. Associate Professor, Pulmonary Medicine Department, Health Sciences University Faculty of Medicine, Atatürk Chest Diseases and Thoracic Surgery Training and Research Hospital, Ankara, Turkey.

**Keywords:** COVID-19, Cytokine release syndrome, Mortality, Interleukins, Neutrophils, Lymphocytes, Tocilizumab supplementary concept, Hyperinflammation, SARS coronavirus 2

## Abstract

**BACKGROUND:**

Coronavirus disease 2019 (COVID-19) can cause cytokine release syndrome (CRS), which leads to high mortality rates. Tocilizumab suppresses CRS by blocking the signal transduction of interleukin-6 (IL-6).

**OBJECTIVE:**

To evaluate the clinical and laboratory parameters associated with mortality among patients receiving tocilizumab treatment.

**DESIGN AND SETTING:**

Retrospective observational study conducted in the chest disease departments of two different training and research hospitals in the center of Ankara, Turkey.

**METHODS:**

Patients who were hospitalized and treated with tocilizumab in September 2020 were retrospectively analyzed. Their laboratory parameters and clinical characteristics were obtained from the hospital information system database. Comparative analyses were performed between the patients who died and the ones who survived.

**RESULTS:**

A total of 58 patients who received tocilizumab treatment were included in this study, among whom 35 (60.3%) died. There was no difference between the mortality and survival groups in terms of white blood cell (WBC), neutrophil, lymphocyte, ferritin or C-reactive protein (CRP) levels detected on admission. WBC, lymphocyte, neutrophil and CRP levels measured on the third and fifth days after tocilizumab administration were found to be significantly lower in the survival group (P < 0.05). In multiple logistic regression analysis, age and oxygen saturation were determined to be independent risk factors for mortality.

**CONCLUSION:**

Persistently high WBC, CRP and neutrophil levels and low lymphocyte levels could be considered to be valuable indicators of mortality among COVID-19 patients treated with tocilizumab. Age and low oxygen saturation are independent risk factors for mortality among patients receiving tocilizumab treatment.

## INTRODUCTION

Coronavirus disease 2019 (COVID-19), which originated in China in December 2019, has become a pandemic affecting the entire world.^1^ It is a matter of grave public health concern because of its rapid spread and associated mortality. COVID-19-associated pneumonia may lead to development of respiratory distress syndrome and, thus, respiratory failure is the most important cause of mortality related to this disease.^2,3^

In the first report of death due to the causative agent of COVID-19, i.e. severe acute respiratory syndrome coronavirus 2 (SARS-CoV-2), it was stated that a pathologically high concentration of proinflammatory cytokines was detected.^4^ Cytokine release syndrome (CRS) can occur because of infection, the action of some drugs or other factors and is characterized by sudden increases in the levels of many proinflammatory cytokines.^5,6^

CRS is more common in diseases related to the immune system, immune system-related treatments such as chimeric antigen receptor T-cell therapy, organ transplant sepsis and viral infections.^7^ Its clinical symptoms can range from a flu-like syndrome to circulatory disorders, pulmonary edema, hypoxia, peripheral edema, hypotension and multiorgan system failure.^6^

Interleukin 6 (IL-6) is an important member of the cytokine system and plays a critical role in acute inflammation, autoimmune cell differentiation and disease treatment.^8^ Tocilizumab is a recombinant, humanized, antihuman IL-6 monoclonal antibody of the immunoglobulin GI (IgG1) subtype. It binds to membrane-bound and soluble IL-6 receptors specifically and provides blockade of signal transduction. It was developed to treat rheumatoid arthritis and systemic juvenile idiopathic arthritis.^9,10^

COVID-19 is severe in approximately 14% and critical in approximately 5% of patients.^11^ This has led to rapid application of various treatments around the world during the pandemic. Many drugs have been tested over the course of treatment of severe COVID-19.

There are a lot of studies in the literature with diverse results regarding the effect of tocilizumab. In an observational study, tocilizumab was shown to contribute to survival.^12^ However, randomized studies on tocilizumab have shown mixed results among patients with varying degrees of severity of COVID-19 and populations with various care standards.^13,14^ Rosas et al. found that clinical status and mortality in the tocilizumab arm of their study were not superior to placebo.^13^ On the other hand, a meta-analysis including six randomized controlled trials showed that tocilizumab treatment reduced the need for mechanical ventilation and/or the rates of all-cause mortality among hospitalized patients.^15^

In a meta-analysis that included 30 studies, older age, male gender, chronic kidney disease, chronic obstructive pulmonary disease, cancer, hypertension, diabetes and laboratory findings such as lymphopenia, thrombocytopenia and high C-reactive protein (CRP), D-dimer, alanine aminotransferase and creatine kinase levels were found to be associated with poor prognosis.^16^ CRP, ferritin, platelet, leukocyte and erythrocyte counts have been recommended as markers showing the severity of hyperinflammation.^15^

In Turkey, severe cases of COVID-19 have always been treated in hospital settings, up to the present day. Therefore, through this study, we planned to evaluate our experiences of administering tocilizumab to patients with severe COVID-19 who were treated outside of the intensive care unit and the clinical characteristics that affected the mortality rate among these patients receiving tocilizumab treatment.

## OBJECTIVE

In this study, we aimed to evaluate the clinical features and laboratory parameters associated with mortality among patients receiving tocilizumab treatment.

## METHODS

Approval for this study was obtained from our hospital’s local ethics committee (approval number and date: 707/31.12.2020). Data were collected retrospectively from the hospital information system database. Patients who were hospitalized and followed up in the department of chest disease at two advanced-level educational and training hospitals, during September 2020, and who received tocilizumab treatment, were eligible for inclusion. Those older than 18 years with COVID-19-positive reverse transcription polymerase chain reaction (RT-PCR) test results and full data availability from the database were included in the study. These patients were divided into two groups: (I) mortality and (II) survival.

Clinical features such as age, gender, body mass index (BMI, kg/m^2^), major comorbidities and symptoms at the time of admission (including coughing, shortness of breath, fever, diarrhea, loss of taste and myalgia) were recorded. Oxygen saturation (sO_2_) and high fever (with body temperature > 38.2 °C) were recorded.

Laboratory parameters such as white blood cell (WBC), lymphocyte, neutrophil, platelet, alanine aminotransferase (ALT), aspartate aminotransferase (AST), creatinine, D-dimer, troponin and CRP levels were recorded. Tocilizumab was administered once at a dose of 8 mg/kg (in accordance with the guidelines of the Ministry of Health^17^), to all the patients. The courses of the WBC, lymphocyte, neutrophil, CRP and ferritin levels measured on the first, third and fifth days after tocilizumab administration were evaluated. Differences in these values between the mortality and survival groups were analyzed.

The chest X-ray findings on admission were classified as normal, unilateral infiltration, or bilateral infiltration. The pathological findings from computed tomography (CT) were categorized as ground glass, consolidation or a combination of these two. In addition, all radiological findings were classified as unilateral, bilateral, central, peripheral or diffuse localization, according to the location. Among CT findings evaluated using axial sections, if ground glass and/or consolidation was detected in less than 25% of all areas, the case was recorded as “mild involvement,” while if this was detected in 25% to 50%, it was recorded as “moderate involvement” and if over 50%, as “severe involvement.” Subsequent radiological findings during the follow-up were not taken into account. Differences were analyzed in terms of radiological findings between the groups.

Treatment protocols were applied in accordance with the guidelines of the Ministry of Health.^17^ Drug treatments and duration, and supportive oxygen treatments (initial and advanced), were recorded. Tocilizumab, high-dose steroid (HDS) and convalescent plasma (CP) were classified and recorded as advanced medical treatments. These advanced treatments were used in patients who did not gain clinical benefit from their initial treatment.

### Statistical analysis

The statistical analysis was performed using SPSS for Windows (version 16.0; SPSS Inc., Chicago, Illinois, United States). A normality analysis on continuous data was conducted using the Shapiro-Wilk test. It was accepted that a P-value of less than 0.05 in the Shapiro-Wilk test did not provide an assumption of normality. For normally distributed variables, the independent-sample t test was used to carry out comparisons of means between pairs of independent groups, and these variables were expressed as means, standard deviations and 95% confidence intervals. For variables that were assumed to be not normally distributed, the Mann-Whitney U-test was used for comparisons between pairs of independent groups, and these variables were expressed as medians and interquartile ranges (IQRs). Chi-square tests were performed to compare the frequency distributions of categorical variables, and these variables were expressed as counts and percentages. Multiple logistic regression analysis was performed for parameters that had been evaluated to be significant, in terms of survival, in univariate analyses. In this analysis, odds ratios were presented with 95% confidence intervals for the potential predictors of mortality. P-values of less than 0.05 were taken to be statistically significant.

## RESULTS

A total of 58 patients who received tocilizumab treatment were included in the study. Of these, 17 (29.3%) were females. Thirty-five (60.3%) of the patients died. The average age was determined to be statistically different between the survival and mortality groups (P = 0.030; 64 [range: 50-68] and 69 [range: 59-72] years, respectively). Although the median BMI was 30 kg/m^2^ (IQR: 28-30) in both groups, there was no difference in terms of BMI. Median sO_2_ on admission was 89% and 85% in the survival and mortality groups, respectively (P = 0.036). There were no statistically significant differences between the two groups in terms of comorbidities and symptoms (Table 1).

**Table 1.** Demographics and symptoms

Variables		Total n = 58 (100.0%)						P-value
				Survival		Mortality		
				n = 23 (39.7%)		n = 35 (60.3%)		
		n (%)	Med (IQR)	n (%)	Med (IQR)	n (%)	Med (IQR)	
Gender	Male	41 (70.7)		16 (39.0)		25 (61.0)		0.879^*^
	Female	17 (29.3)		7 (41.2)		10 (58.8)		
Age			66.5 (57-71)		64 (50-68)		69 (59-72)	0.030**
BMI			30 (28-30)		30 (28-32)		29 (27.8-30)	0.196**
Admission sO_2_		86.5 (75-90)		89 (80-91)		85 (75-89)		0.036**
BT > 38.2 °C		9 (15.5)		1 (11.1)		8 (88.9)		0.073***
Comorbidity		43 (74.1)		16 (37.2)		27 (62.8)		0.519^*^
HT		36 (62.1)		13 (36.1)		23 (63.9)		0.480^*^
DM		19 (32.8)		9 (47.4)		10 (52.6)		0.402^*^
CAD		12 (20.7)		3 (25.0)		9 (75.0)		0.329***
CHF		3 (5.2)		1 (33.3)		2 (66.7)		1.000***
COPD		7 (12.1)		2 (28.6)		5 (71.4)		0.692***
Asthma		3 (5.2)		0 (0.0)		3 (100.0)		0.270***
Malignancy		0 (0.0)		0 (0.0)		0 (0.0)		–
CKD		1 (1.7)		0 (0.0)		1 (100.0)		1.000***
ILD		0 (0.0)		0 (0.0)		0 (0.0)		–
Rheumatological		2 (3.4)		0 (0.0)		2 (100.0)		0.513***
Cough		54 (93.1)		21 (38.9)		33 (61.1)		1.000***
Dyspnea		58 (100.0)		23 (39.7)		35 (60.3)		–
Sore throat		30 (51.7)		11 (36.7)		19 (63.3)		0.630^*^
Fever		20 (34.5)		8 (40.0)		12 (60.0)		0.969^*^
Diarrhea		7 (12.1)		1 (14.3)		6 (85.7)		0.226***
Taste-smell disorder		8 (13.8)		2 (25.0)		6 (75.0)		0.458***
Myalgia		30 (51.7)		9 (30.0)		21 (70.0)		0.120^*^

Med = median; IQR = interquartile range; BMI = body mass index; sO_2_ = oxygen saturation; BT = body temperature; C = Celsius; HT = hypertension; DM = diabetes mellitus; CAD = coronary artery disease; CHF = congestive heart failure; COPD = chronic obstructive pulmonary disease; CKD = chronic kidney disease; ILD = interstitial lung disease. * chi-square test; **Mann-Whitney U test; ***Fisher’s exact test.

**Figure 1.**
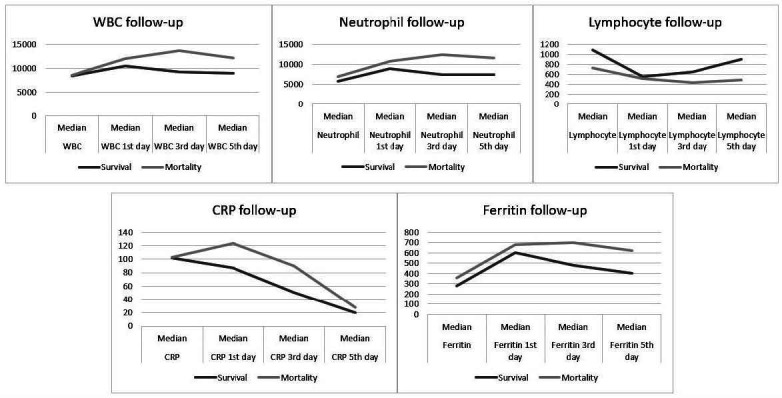
Predictors levels during follow-up. WBC = white blood cell; CRP = C-reactive protein.

There were no differences between the two groups in terms of WBC, neutrophil, lymphocyte, AST, ALT, D-dimer, ferritin, troponin or CRP levels. However, a significant difference was found between the survival and mortality groups regarding the WBC, lymphocyte, neutrophil and CRP levels detected on the third and fifth days after tocilizumab treatment (P < 0.05; Table 2). The course of WBC, neutrophil, lymphocyte, CRP and ferritin levels over the days after tocilizumab treatment is shown in Figure 1. There were no differences between the groups in terms of the chest X-ray and CT findings (Table 3).

**Table 2.** Laboratory test data

	Total			P-value
		Survival	Mortality	
	Median (IQR) or Mean ± SD	Median (IQR) or Mean ± SD	Median (IQR) or Mean ± SD	
WBC	8455 (5690-10560)	8430 (6206-10420)	8480 (5580-10880)	0.733^*^
WBC 1^st^ day	10800 (8910-14580)	10420 (7680-13230)	11925 (9070-15115)	0.408^*^
WBC 3^rd^ day	12108 ± 5400	10264 ± 4729	13476 ± 5530	0.029^†^
WBC 5^th^ day	11681 ± 6127	9275 ± 4866	13484 ± 6429	0.016^†^
Lymphocyte	840 (570-1220)	1080 (690-1660)	720 (490-980)	0.025^*^
Lymphocyte 1^st^ day	601 ± 321	731 ± 387	507 ± 226	0.018^†^
Lymphocyte 3^rd^ day	525 (380-860)	650 (450-1100)	440 (350-580)	0.005^*^
Lymphocyte 5^th^ day	620 (460-930)	900 (780-1200)	485 (415-670)	< 0.001^*^
Neutrophil	6625 (3890-8940)	5700 (3600-7420)	6990 (3900-9050)	0.262^*^
Neutrophil 1^st^ day	10013 ± 3718	9240 ± 4008	10568 ± 3452	0.194^†^
Neutrophil 3^rd^ day	10920 ± 5231	8983 ± 4437	12357 ± 5375	0.018^†^
Neutrophil 5^th^ day	10245 ± 5885	7450 ± 3738	12340 ± 6371	0.002^†^
Monocyte	445 (300-660)	540 (350-720)	370 (270-580)	0.107^*^
Eosinophil	1 (0-1)	1 (0-10)	0 (0-1)	0.193^*^
Hemoglobin	13.6 ± 1.6	13,9 ± 1,4	13,5 ± 1,6	0.319^†^
Platelet	228000 (176000-273000)	238000 (182000-305000)	226000 (168000-273000)	0.645^*^
Creatinine	0.91 (0.74-1.1)	0.87 (0.75-1.1)	0.93 (0.73-1.1)	0.679^*^
ALT	35 (24-50)	30 (22-56)	36 (25-49)	0.460^*^
AST	43 ± 19	41 ± 16	45 ± 20	0.387^†^
Na	137 (135-139)	137 (134-139)	137 (135-140)	0.949^*^
Ca	8.8 (8.5-9)	8.8 (8.4-8.9)	8.8 (8.5-9)	0.774^*^
D-dimer	0.6 (0.4-0.7)	0.5 (0.3-0.7)	0.6 (0.4-0.9)	0.289^*^
Troponin	8.7 (5.6-16.2)	8.5 (4.4-12.6)	9 (6.4-17)	0.195^*^
CRP	102.5 (48.5-143)	102 (44.9-144)	103 (69-139)	0.899^*^
CRP 1^st^ day	111.7 ± 49.4	93.4 ± 42.4	124.8 ± 50.4	0.018^†^
CRP 3^rd^ day	65 (40-100)	50 (22-85)	90 (51-118)	0.019^*^
CRP 5^th^ day	21 (13-61)	20 (10-26)	27.5 (16.5-86.5)	0.031^*^
CRP TOCI day	177.8 ± 62.2	155.3 ± 53.4	192.6 ± 63.7	0.024^†^
Ferritin	311.3 (88-536)	280 (124-432)	357 (76.9-595.3)	0.874^*^
Ferritin 1^st^ day	615 (350-1250)	600 (234-1036)	678 (400-1387)	0.656^*^
Ferritin 3^rd^ day	599 (350-1100)	480 (345-869)	700 (389-1200)	0.278^*^
Ferritin 5^th^ day	504 (333-1133)	400 (333-699)	621.5 (361.5-1300)	0.074^*^

IQR = interquartile range; SD = standard deviation; WBC = white blood cell; ALT = alanine aminotransferase; AST = aspartate aminotransferase; Na: sodium; Ca = calcium; CRP = C-reactive protein; TOCI = tocilizumab. * Mann-Whitney U test; median (IQR); ^†^independent-sample t test; mean ± SD.

**Table 3.** Imaging data

Variables		Total			P-value
			Survival	Mortality	
		n (%)	n (%)	n (%)	
Chest X-ray	None	7 (12.1)	3 (42.9)	4 (57.1)	0.758*
	Unilateral	13 (22.4)	4 (30.8)	9 (69.2)	
	Bilateral	38 (65.5)	16 (42.1)	22 (57.9)	
CT type	HRCT	7 (12.5)	4 (57.1)	3 (42.9)	0.429^†^
	Thorax CT	49 (87.5)	19 (38.8)	30 (61.2)	
CT signs	Ground glass	32 (57.1)	10 (31.3)	22 (68.8)	0.051*
	Consolidation	21 (37.5)	10 (47.6)	11 (52.4)	
	Ground glass + Consolidation	3 (5.4)	3 (100.0)	0 (0.0)	
Lobe involvement	< 25%	19 (33.9)	8 (42.1)	11 (57.9)	0.823*
	25-50%	29 (51.8)	11 (37.9)	18 (62.1)	
	> 50%	8 (14.3)	4 (50.0)	4 (50.0)	
CT unilateral/bilateral	Unilateral	2 (3.6)	2 (100.0)	0 (0.0)	0.164^†^
	Bilateral	54 (96.4)	21 (38.9)	33 (61.1)	
CT lobes	Upper lobes	2 (3.6)	0 (0.0)	2 (100.0)	0.482*
	Lower lobes	31 (55.4)	13 (41.9)	18 (58.1)	
	All	23 (41.1)	10 (43.5)	13 (56.5)	
CT central/peripheral	Central	0 (0.0)	0 (0.0)	0 (0.0)	0.183^‡^
	Peripheral	35 (62.5)	12 (34.3)	23 (65.7)	
	Diffuse	21 (37.5)	11 (52.4)	10 (47.6)	

CT = computed tomography; HRCT = high-resolution computed tomography. *chi-square test (insufficient sample counts); ^†^Fisher’s exact test; ^‡^chi-square test.

The initial treatment modalities are presented in Table 4. At the beginning, nine (15.5%) of the patients did not receive any steroids. Dexamethasone was administered at a dose of 8 mg/day and methylprednisolone at a dose of 40 or 80 mg/day. However, methylprednisolone was given at a high dose of 250 mg/day for three days to three patients (Table 4). There were no significant differences between the survival and mortality groups in terms of broad-spectrum antibiotics, low-molecular-weight heparin (LMWH), supportive oxygen treatments or the time of transition to advanced treatments (P > 0.05; Table 4).

**Table 4.** Treatment and follow-up

Variables		Total						P-value
				Survival		Mortality		
		n (%)	Median (IQR) or Mean ± SD	n (%)	Median (IQR) or Mean ± SD	n (%)	Median (IQR) or Mean ± SD	
Hospitalization duration			20 (15-29)		23 (18-30)		20 (14-25)	0.063*
Plaquenil		43 (74.1)		15 (34.9)		28 (65.1)		0.208^†^
Favipiravir		56 (96.6)		23 (41.1)		33 (58.9)		0.513^‡^
Steroid	None	9 (15.5)		6 (66.7)		3 (33.3)		0.096^†^
	Dexamethasone	35 (60.3)		14 (40)		21 (60)		
	M-prednisolone	14 (24.1)		3 (21.4)		11 (78.6)		
LMWH		58 (100)		23 (39.7)		35 (60.3)		–
Antibiotics		57 (98.3)		23 (40.4)		34 (59.6)		1.000^‡^
C-vit		39 (67.2)		14 (35.9)		25 (64.1)		0.402^†^
Dexamethasone dose			8 (0-8)		8 (0-8)		8 (0-8)	0.780*
M-prednisolone dose (mg/day)	0	39 (67.2)		18 (46.2)		21 (53.8)		0.294**
	40	6 (10.3)		0 (0)		6 (100)		
	80	10 (17.2)		3 (30)		7 (70)		
	250	3 (5.1)		2 (66.7)		1 (33.3)		
Wide-spectrum AB	None	2 (3.4)		1 (50)		1 (50)		0.700^§^
	Admission	24 (41.4)		8 (33.3)		16 (66.7)		
	Follow-up	32 (55.2)		14 (43.8)		18 (56.3)		
AB change time			5 (5-7)		5 (5-7)		5 (4-7)	0.526*
Initial O_2_ therapy	None	10 (17.2)		8 (80)		2 (20)		0.174**
	Nasal	26 (44.8)		9 (34.6)		17 (65.4)		
	Venturi-reservoir	14 (24.1)		3 (21.4)		11 (78.6)		
	HF	8 (13.8)		3 (37.5)		5 (62.5)		
Advanced O_2_ therapy	None (additional)	26 (44.8)		11 (42.3)		15 (57.7)		0.804^†^
	Venturi-reservoir	14 (24.1)		6 (42.9)		8 (57.1)		
	HF/CPAP	18 (31)		6 (33.3)		12 (66.7)		
O_2_ therapy change time			5 ± 2		5 ± 2		5 ± 2	0.304^¶^
Therapy change time			5 (3-6)		5 (3-7)		5 (4-6)	0.846*
Advanced medical therapy	Toci	7 (12.1)		1 (14.3)		6 (85.7)		0.311^§^
	Toci + HDS	2 (3.4)		1 (50)		1 (50)		
	Toci + plasma	48 (82.8)		20 (41.7)		28 (58.3)		
	Toci + HDS + plasma	1 (1.7)		1 (100)		0 (0)		

IQR = interquartile range; SD = standard deviation; M = methyl; AB = antibiotic; LMWH = low-molecular-weight heparin; O_2_ = oxygen; HF = high-flow oxygen therapy; CPAP = continuous positive airway pressure; Toci = tocilizumab; HDS = high-dose steroid; *Mann-Whitney U test; ^†^chi-square test; ^‡^Fisher’s exact test; ^§^chi-square test (insufficient sample counts); ^¶^independent-sample t test; **Bonferroni correction used in the analysis on multiple groups, with adjusted P-values.

With regard to advanced medical treatments, seven patients (12.1%) received only tocilizumab, two (3.4%) received tocilizumab and HDS, 48 (82.8%) received tocilizumab and CP, and one received tocilizumab, HDS and CP. There were no differences between the groups in terms of these treatment modalities (P > 0.05).

The mortality rate was found to be higher among patients with initial supportive oxygen treatment, such as a Venturi/reservoir mask or high-flow nasal oxygen (78.6% and 62.5%, respectively). Increasing the oxygen support was not found to be associated with mortality during the follow-up (P = 0.804; Table 4).

Age and sO_2_ levels on admission were found to be independent risk factors for mortality in the multiple logistic regression analysis (Table 5).

**Table 5.** Multiple logistic regression analysis

		B	Sig.	Exp(B)
Step 1a	Age	0.080	0.014	1.083 (1.016-1.154)
	sO_2_ (admission)	-0.063	0.048	0.939 (0.882-1.000)
	Constant	0.567	0.850	1.764

sO_2_ = oxygen saturation; ^a^variable(s) entered in step 1: age and sO_2_ (admission); Sig. = significance. Equation: logit (P) = 0.567 + (0.08 x age) + (-0.063 x sO_2_).

## DISCUSSION

There are still many uncertainties surrounding COVID-19 treatment, and differing results have been obtained among patients treated with tocilizumab. The aim of this study was to investigate clinical features that might predict different outcomes from tocilizumab treatment among similar patients. It was found that some inflammation markers could be used for such predictions.

One of the cytokines responsible for CRS is IL-6. Cardio- myopathy, complement activation, coagulation cascade activation and hyperinflammation-like disseminated intravascular coagulation develop because of IL-6 release.^18^ Tocilizumab reduces the harmful effects of hyperinflammation by decreasing IL-6 signal transmission in cases of severe CRS.^19^ Therefore, patients diagnosed with COVID-19 should be followed up regarding hyperinflammation.^20^

In this study, there were no significant differences between the mortality and survival groups in terms of WBC, neutrophil, CRP, D-dimer or ferritin levels. However, significantly higher CRP, WBC and neutrophil levels and lower lymphocyte levels were detected in the mortality group on the first, third and fifth days after tocilizumab administration. Therefore, it can be thought that the course followed by inflammation markers after treatment may be associated with mortality.

In one study, ferritin was shown to be a marker for macrophage activation syndrome.^20^ In another study involving 150 patients, ferritin and IL-6 levels (which are inflammatory factors) were found to be associated with mortality.^21^ In the current study, no significant difference in ferritin levels was found between the mortality and survival groups. However, looking at the first, third and fifth days of follow-up after tocilizumab treatment, there were persistent high ferritin levels in the mortality group (Figure 1). Considering that all the patients had severe COVID-19, no difference was expected between the two groups. Therefore, persistently high ferritin levels during the follow-up may be an indicator of poor prognosis.

In a study conducted on 21 patients in China, it was found that lymphocyte and CRP levels returned to normal after tocilizumab treatment. After tocilizumab administration, the need for oxygen support decreased in 15 of the patients, and there was no need for oxygen support in one.^14^ In the current study, a statistically significant decrease in CRP levels was observed in the survival group after tocilizumab treatment. CRP is thought to be a suitable surrogate marker that reflects IL-6 bioactivity. In another study on 15 cases, a significant decrease in CRP levels was seen after tocilizumab treatment.^22^ This suggests that mortality was lower in the group in which the IL-6 effect decreased faster after tocilizumab treatment.

In a study on 63 severe cases of COVID-19, the patients received intravenous and subcutaneous tocilizumab, and no difference between these drug administration methods was found after treatment. In addition, improvements in CRP, ferritin, D-dimer and lymphocyte levels were observed in all 63 patients.^23^

The decrease in lymphocyte levels in COVID-19 is an important marker for diagnosis and disease severity.^24^ In a meta-analysis, it was reported that tocilizumab treatment had no effect on lymphocyte and neutrophil levels.^25^ However, in the current study, lower lymphocyte levels on admission and in the follow-up during tocilizumab treatment were detected in the mortality group. Although an improvement in lymphocyte levels after tocilizumab treatment was observed in the mortality group, these levels were found to still be low.

Zhao et al. reviewed 13 retrospective studies and reported that use of tocilizumab significantly reduced mortality rates, compared with standard therapy (odds ratio, OR = 0.44; 95% confidence interval, CI: 0.36-0.55).^25^ In another study, it was stated that use of tocilizumab in severe cases of COVID-19 was safe and had a positive effect regarding improvement of many laboratory parameters.^26^ In a meta-analysis that examined the causal relationships between interventions and outcomes and included reference randomized controlled studies, it was stated that administering tocilizumab to patients who had been hospitalized due to COVID-19 did not reduce the risk of all-cause mortality in these patients, but it reduced the possibility of needing mechanical ventilation.^15^

In our study, the mortality rate was 60.3%, but all the patients were diagnosed with severe COVID-19. Among the hospitalized patients who needed a Venturi mask and high-flow oxygen, the resultant mortality rates were 78.6% and 62.5%, respectively. These data corroborated that notion that tocilizumab administration has no effect on mortality among patients using mechanical ventilation, as determined in the meta-analysis.^25^

In our study, no difference in radiological signs in either chest X-rays or computed tomography (CT) images was found between the survival and mortality groups. It can be said that the patients were a homogeneous group in terms of their radiological involvement. Therefore, it was not logical to evaluate the relationship between radiological findings and mortality in this study.

There were several limitations to our study. First, it was retrospective and had a limited number of patients. Second, IL-6 levels could not be studied in these patients in relation to hospital conditions.

Furthermore, the patients were recruited from two different hospitals. The Ministry of Health treatment guidelines were used in both hospitals, but the optional treatment approaches, especially high-dose steroids and convalescent plasma, were administered according to the individual preferences of the doctors. Lastly, all the patients who received tocilizumab treatment were severely ill, and the lack of comparison with a control group that did not receive tocilizumab can be considered to be a limitation. However, tocilizumab was used according to its availability during the pandemic and, therefore, it was not possible to compare the laboratory parameters of the group not given tocilizumab with those of patients with similar disease severity.

Through prospective studies in which the severity of the disease can be scored in terms of clinical, radiological and laboratory parameters, treatment arms can be better standardized and changes in all parameters after treatment can be revealed more clearly.

## CONCLUSION

A few laboratory findings that could predict mortality among COVID-19 patients receiving tocilizumab treatment were detected. High WBC, neutrophil and CRP levels and persistently low lymphocyte levels may be indicators of mortality. In addition, age (> 65 years) and low sO_2_ on admission are independent risk factors for mortality among patients receiving tocilizumab treatment. However, we need to state that, because of the retrospective design of our study, selection of the patients for tocilizumab treatment may have affected the results.
